# Functional Characterization and Phenotyping of Protoplasts on a Microfluidics-Based Flow Cytometry

**DOI:** 10.3390/bios12090688

**Published:** 2022-08-26

**Authors:** Xingda Dai, Shuaihua Zhang, Siyuan Liu, Hang Qi, Xuexin Duan, Ziyu Han, Jiehua Wang

**Affiliations:** 1 School of Environmental Science and Engineering, Tianjin University, Weijin Rd. 92, Tianjin 300072, China; 2 State Key Laboratory of Precision Measuring Technology and Instruments, College of Precision Instruments and Optoelectronics Engineering, Tianjin University, Weijin Rd. 92, Tianjin 300072, China

**Keywords:** microfluidic flow cytometry, protoplasts, fluorescent reporter, UV lights, anthocyanidin, primary cell wall

## Abstract

A better understanding of the phenotypic heterogeneity of protoplasts requires a comprehensive analysis of the morphological and metabolic characteristics of many individual cells. In this study, we developed a microfluidic flow cytometry with fluorescence sensor for functional characterization and phenotyping of protoplasts to allow an unbiased assessment of the influence of environmental factors at the single cell level. First, based on the measurement of intracellular homeostasis of reactive oxygen species (ROS) with a DCFH-DA dye, the effects of various external stress factors such as H_2_O_2_, temperature, ultraviolet (UV) light, and cadmium ions on intracellular ROS accumulation in Arabidopsis mesophyll protoplasts were quantitatively investigated. Second, a faster and stronger oxidative burst was observed in Petunia protoplasts isolated from white petals than in those isolated from purple petals, demonstrating the photoprotective role of anthocyanins. Third, using mutants with different endogenous auxin, we demonstrated the beneficial effect of auxin during the process of primary cell wall regeneration. Moreover, UV-B irradiation has a similar accelerating effect by increasing the intracellular auxin level, as shown by double fluorescence channels. In summary, our work has revealed previously underappreciated phenotypic variability within a protoplast population and demonstrated the advantages of a microfluidic flow cytometry for assessing the in vivo dynamics of plant metabolic and physiological indices at the single-cell level.

## 1. Introduction

Complex biological processes rely on the dynamic behavior of single cells and cell–cell interactions. Conventional bulk tissue analysis obscures differences in cell diversity in most biological/biomedical samples, whereas single-cell analysis allows a qualitative and/or quantitative characterization of each cell in a cell population. It not only reveals biological variability between cell populations, but also paves the way to new insights into various cellular processes such as cell-fate decisions, physiological heterogeneity, or genotype–phenotype linkages [1,2].

With respect to single-cell phenotyping, flow cytometry has demonstrated its ability for high-throughput quantitative analysis and isolation of targeted biological samples. However, conventional flow cytometry is bulky, complex, and requires highly skilled personnel. With the development of microfluidic technology, microfluidic flow cytometry (MFCM) has been combined with flow cytometry to achieve powerful single cell focusing, detection, and sorting, which has been proven in various biological applications [3], including single-cell RT-PCR [4], stem cell screening [5], protein analysis [6], etc. While MFCM has proven to be a powerful tool for single-cell manipulation and analysis in medical diagnostics and animal cell studies, similar work on plant-cell properties is still far behind.

In this study, we developed a microfluidic flow cytometry that provides a simple, direct, and cost-effective solution for the analysis of protoplast samples. Protoplasts are plant cells in which the cell wall has been enzymatically or mechanically removed [7], and they are a very efficient experimental model for biotechnological applications such as somatic hybridization and genetic transformation. Protoplasts offer many cytological advantages that multicellular tissues and cell assemblies in suspension culture do not and are therefore an invaluable experimental system for studying cellular processes such as signal transduction [8], cell wall regeneration [9], the role of stress and hormones [10], and transient gene expression [11]. However, after cell wall digestion, the resulting protoplasts are osmosis-sensitive, fragile structures that require extreme care to maintain their integrity. In addition, protoplasts are generally larger in diameter than mammalian cells and do not adhere like animal cells, so analysis of protoplast populations using flow cytometry requires significant changes in instrument configuration, and is extremely difficult to manage to achieve stable flow [12].

To investigate the performance of the developed system, we first examined Arabidopsis mesophyll protoplasts for their intracellular accumulation of reactive oxygen species (ROS) in response to a variety of external stimuli. In plant cells, ROS usually refer to superoxide radicals (O_2_^•−^), hydrogen peroxide (H_2_O_2_), and hydroxyl radicals (^•^OH), and as in animal cells, when in excess they are generally believed to have deleterious effects on cell metabolism, leading to cell dysfunction and death [13,14]. In plants, ROS can be triggered by a number of abiotic and biotic stresses [14,15], such as bright light, cold, and desiccation [16,17,18], and plants have developed a variety of antioxidant strategies to scavenge ROS based on enzymatic reactions and the direct radical scavenging property of non-enzymatic antioxidants such as ascorbate and glutathione [18,19]. On the other hand, ROS also function as a signaling molecule mediating the induction and/or response to stress [20,21,22,23]. For example, H_2_O_2_ regulates stomatal movement and plant–pathogen interactions [14,24], and both O_2_ and H_2_O_2_ can trigger a range of defense responses [23,25]. Therefore, the effects of ROS vary with concentration, with high concentrations leading to hypersensitive cell death [26], while low concentrations promote cell cycle progression and secondary cell wall differentiation [27]. In this work, we used dichlorodihydrofluorescein diacetate (DCFH-DA) fluorescence biosensors to determine the intracellular ROS dynamics in plant cells. In addition to the responses of mesophyll protoplasts to various environmental stresses, we also compared the ROS responses of non-photosynthetic protoplasts isolated from Petunia petal tissue to ultraviolet-A (UV-A) and UV-B irradiation and validated the long-standing question of the photoprotective effects of anthocyanins. As another example of the functional phenotyping of single plant cells, we examined the regulatory function of auxin, an important phytohormone, in the process of primary cell wall regeneration using wild-type and genetically deficient protoplasts. We also demonstrated the correlation between the promotion of primary cell wall regeneration by UV irradiation and the regulation of intracellular auxin by dual-channel fluorescence detection. Our data presented here are an example of the use of microfluidic flow cytometry in single-cell plant research and extend our understanding of two plant-specific processes at the cellular level. This system allowed a more accurate and efficient assessment of cell status, and an unbiased interpretation of the average behavior of single plant cells. More importantly, the results obtained demonstrate that due to the obvious physiological heterogeneity within a plant cell population, a manual representative fluorescence photograph of a single cell can lead to bias and a non-objective or ambiguous interpretation of the experimental results. Understanding cellular function in this post-genomic era is a great opportunity and challenge. We hope that as microfluidic technologies become more advanced and readily available, new microfluidic systems will also undergo significant development and innovation in plant cytology.

## 2. Methods

### 2.1. Device Fabrication and Optical Detection Setup

The microfluidic device was fabricated in poly(dimethyl-siloxane) (PDMS) using soft lithography [28], which had a channel height of 60 μm, a channel width of 40 μm, and holes that were punched for channel inlets and outlets. The fluorescent signals of single plant cells were measured using a microfluidic flow cytometry. The measurement was set up on an inverted microscope (IX73, Olympus, Tokyo, Japan) using 50 mW/360 nm and 50 mW/488 nm lasers as excitation light sources coupled into a beam by a dichroic filter (MD416, Thorlabs Inc., Newton, NJ, USA). The intensity of the two lasers was adjusted using the variable metallic neutral density filters (NDC-50C-2M-A, Thorlabs Inc., Newton, NJ, USA). A transmission light source (850 nm) was mounted on top of the microfluidic device for athe camera’s real-time imaging. The fluorescence emission light and transmission light were split by two dichroic, long-pass filters (DMLP490R and DMLP567R, Thorlabs Inc., Newton, NJ, USA) and collected through a side port of the microscope using two PMTs (H10722-210, HAMAMATSU Photonics, Hamamatsu, Japan) for the simultaneous acquisition of two different colors, and one high-speed camera (UX50, Photron, Tokyo, Japan). LabVIEW 2016 software on a PC with a FPGA data acquisition card (PCIe-7842R, National Instruments, Austin, TX, USA) was applied to record the real-time output voltage of the two PMTs which revealed the fluorescent intensities of single cells. The peak values were then extracted by a MATLAB data processing program and normalized by saturation voltage (5 V) as the relative fluorescence units (RFU) for data analysis.

### 2.2. Protoplast Isolation and Disposal

The *Arabidopsis* Columbia-0 (Col-0) ecotype, the auxin excess mutant *sur2*, the auxin deficient mutant *taa1*, the *DRF-GFP* transgenic strain that responds to auxin signals with spontaneous green fluorescent, and the white and purple flowers of Petunia were used in this study. The plants were grown in a greenhouse at 22 °C under fluorescent white light with a 16-h light/8-h dark cycle. The method for isolation of Arabidopsis leaf protoplasts was obtained with a slight modification of the method of Yoo et al. [11]. In brief, healthy and fully expanded Arabidopsis leaves were washed, the lower epidermis was torn off and then transferred to the enzyme solution (1.5% cellulase R10, 0.4% macerozyme R10, 0.4 M mannitol, 20 mM KCl, and 10 mM MES at pH 5.8). Before use, this enzyme solution was warmed at 55 °C for 10 min, then cooled down to room temperature and combined with CaCl_2_ to 10 mM and BSA to 0.1%, and incubated in darkness at 26 °C for at least 3 h. After enzymatic digestion, an equal volume of W5 solution (154 mM NaCl, 125 mM CaCl_2_, 5 mM KCl, and 2 mM MES at pH 5.8) was used to stop the enzymatic digestion. The mixture was then filtered through a 75-μm nylon mesh into a 50-mL round-bottom tube. After centrifugation at 100× *g* for 6 min, the protoplasts were resuspended by gentle swirling in a culture medium (0.32% B-5 medium, 0.25 M mannitol, and 4 mM MES at pH 5.8). After repeated centrifugation with the culture medium, the protoplasts were resuspended in the culture medium, and the concentration was determined to be approximately 10^6^ cells/mL by hemocytometer. The protoplast extraction from Petunia was modified according to the method of Kang et al. [29]. Fresh flowers were gently rinsed, cut into thin strips and transferred to an enzymatic digestion solution (4.5% cellulase R10, 1.2% macerozyme R10, 0.6 M mannitol, 10 mM CaCl_2_, and 20 mM MES at pH 5.8), and the process after enzymatic digestion was similar to that of Arabidopsis, except that the culture medium was replaced with a buffer (0.6 M mannitol, 10 mM CaCl_2_, and 20 mM MES at pH 5.8).

The protoplasts were transferred to culture plates with 24 wells and placed at temperatures (4, 20, and 37 °C), under UV irradiation (UVA, UVB), at different concentrations of H_2_O_2_ (0, 50, 100, 200, 400, 600, and 800 μM), Cd^2+^ (40 μM), and IAA (5.7 μM) according to the experimental design.

### 2.3. Fluorescence Intensity Observation

The ROS of protoplasts were measured with the fluorescent probe DCFH-DA (S0033M, Beyotime Biotechnology Inc., Shanghai, China), and the protoplast-regenerated primary cell walls were stained with the fluorescent brightener agent Calcofluor white [30]. The different biological responses of protoplasts were qualitatively characterized by fluorescence images taken with a camera (DS-Fi1c, Nikon, Tokyo, Japan) mounted on a microscope (Eclipse 50i, Nikon, Tokyo, Japan) in fluorescence mode (excitation: 400 nm, emission: 450 nm; excitation: 488 nm, and emission: 525 nm).

### 2.4. ROS Localization and Flavonoid Detection in Plant Tissues

Plant tissues were completely immersed in a DAB staining solution (1 mg/mL, pH 3.8) or NBT staining solution (NBT powder was dissolved in 50 mM sodium phosphate solution and prepared to a concentration of 2 mg/mL, pH 7.5) and reacted for 12 h at room temperature in the dark to locate H_2_O_2_ or O_2_^•−^. The plant tissues were then immersed in anhydrous ethanol and heated in a boiling-water bath until the original color was removed so that the stain was clearly visible [31,32]. The determination of total flavonoids and anthocyanins was based on the method of Lin et al. and Hosu et al. [33,34]. All unlabeled chemicals were from Sigma-Aldrich, with the exception of Cellulase R10 and Macerozyme R10, which are purchased from Yakult Pharmaceutical Ind. Co., Ltd. (Tokyo, Japan).

## 3. Results and Discussion

### 3.1. System Setup and Monitoring of Intracellular Redox Status of Mesophyll Protoplast

**Figure 1:**
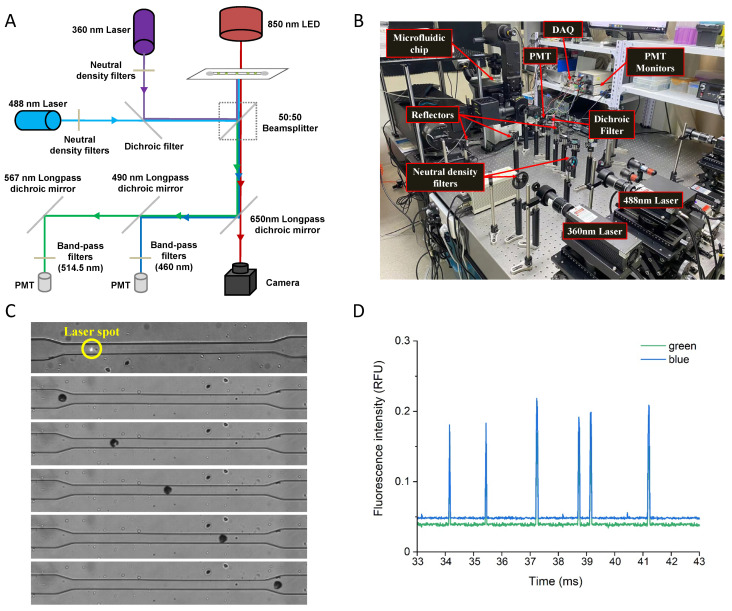
Microfluidic flow cytometry. (**A**) Schematic diagram of the developed platform; (**B**) Photograph of the developed platform; (**C**) Time-lapse images of a single plant cell passing through the channel; (**D**) Real-time response of dual-channel fluorescence detection of a single plant cell.

**Figure 2:**
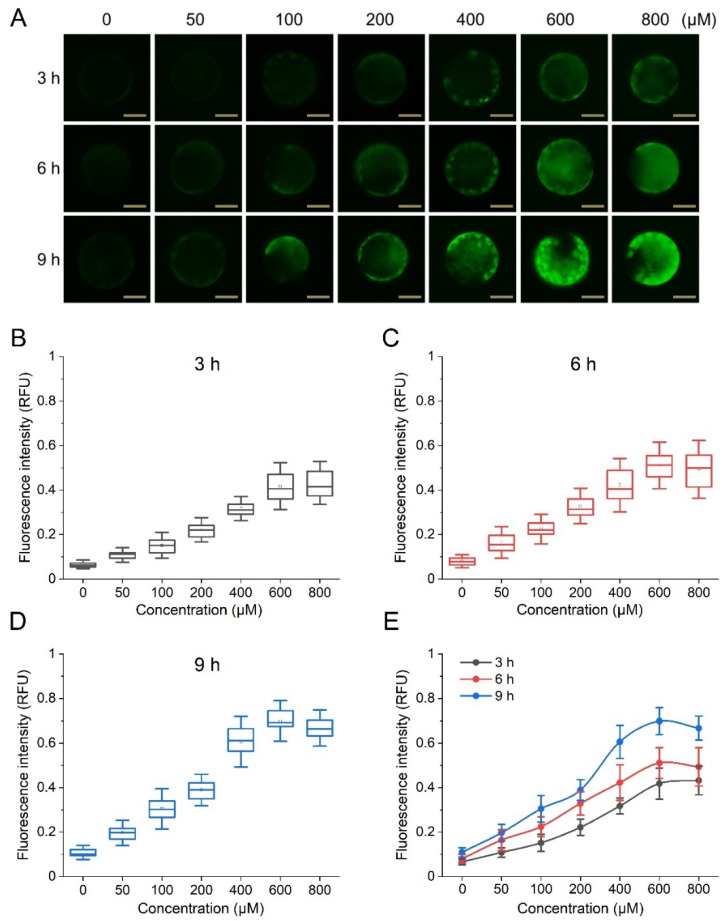
Exogenous H_2_O_2_-mediated changes in ROS content of Arabidopsis protoplasts. (**A**) Fluorescence images of protoplasts with a scale of 25 μm; (**B**–**D**) fluorescence intensity gradients induced by H_2_O_2_ concentration in protoplasts after 3, 6, and 9 h, respectively; (**E**) effect of H_2_O_2_ treatment time on the fluorescence intensity of protoplasts.

To date, cytosolic ROS concentrations of protoplasts measured with fluorescent probes have often been determined empirically on representative cells, which carries the risk that they do not reflect the average value, and do not allow reliable conclusions to be drawn [23,35]. To address this problem and reveal heterogeneity between cell populations, we developed a microfluidic flow cytometry with an integrated fluorescence detector (Figure 1A,B) that allows cell analysis at a theoretical rate of 1000 cells per second. In this system, the protoplast suspension was injected into the microfluidic device at a constant flow rate under controlled pressure (Fluigent MFCS-EZ, pressure ranges 0–1 bar and 0–345 mbar). Because single cells move rapidly through a microfabricated constriction channel whose width corresponds to the cell size (Figure 1C), the real-time output fluorescence signals from the dual-channel PMTs were recorded simultaneously using a LabVIEW program via a data acquisition card (DAQ) (Figure 1D). The acquisition frequency of the DAQ was set at 100 kHz to maintain sampling accuracy and optimize storage efficiency. The protoplast suspension was run at a flow rate of approximately 1 μL/s to maintain a balance between relatively high flow rates and low sheath-fluid pressure. To evaluate the feasibility of this system, we first tested it on isolated Arabidopsis mesophyll protoplasts and exposed them to various concentrations of H_2_O_2_ for 3, 6 and 9 h before adding 2,7-dichlorodihydrofluorescein diacetate (DCFH-DA), a cell-permeable, nonfluorescent probe. During the subsequent 5 min incubation in the dark, the cell-permeable DCFH-DA probe penetrates the protoplast and preferentially distributes in the cytoplasm [23,35], leading to rapid production of the oxidized fluorescence products in the presence of the cellular ROS corresponding to the synchronous peak in the green channel (514.5 nm). As shown in Figure 2, under the present experimental conditions, exogenously applied H_2_O_2_ led to a progressive increase in ROS accumulation within protoplasts in a time- and dose-dependent manner. Concentrations of less than 600 μM H_2_O_2_ showed good linear agreement in terms of the intensity of the intracellular ROS signal; however, at a higher concentration of 800 μM, there was no further increase in intracellular fluorescence intensity, suggesting that cells may turn on specific quenching mechanisms to detoxify and remove the reactive intermediates (Figure 2). This result was essentially in agreement with a previous report that when an onion surface was treated with H_2_O_2_, saturation of the fluorescence response was observed at 1 mM [36]. More importantly, our data show that with the reliable and rapid acquisition of the fluorescence intensity of each cell in the population, the complete data set for the entire population can characterize the properties of the cell population with higher accuracy, and circumvent the potential bias and time waste of conventional microscopy in selecting representative cells.

### 3.2. Cytosolic Redox Status of Mesophyll Protoplast upon Environmental Stresses

**Figure 3:**
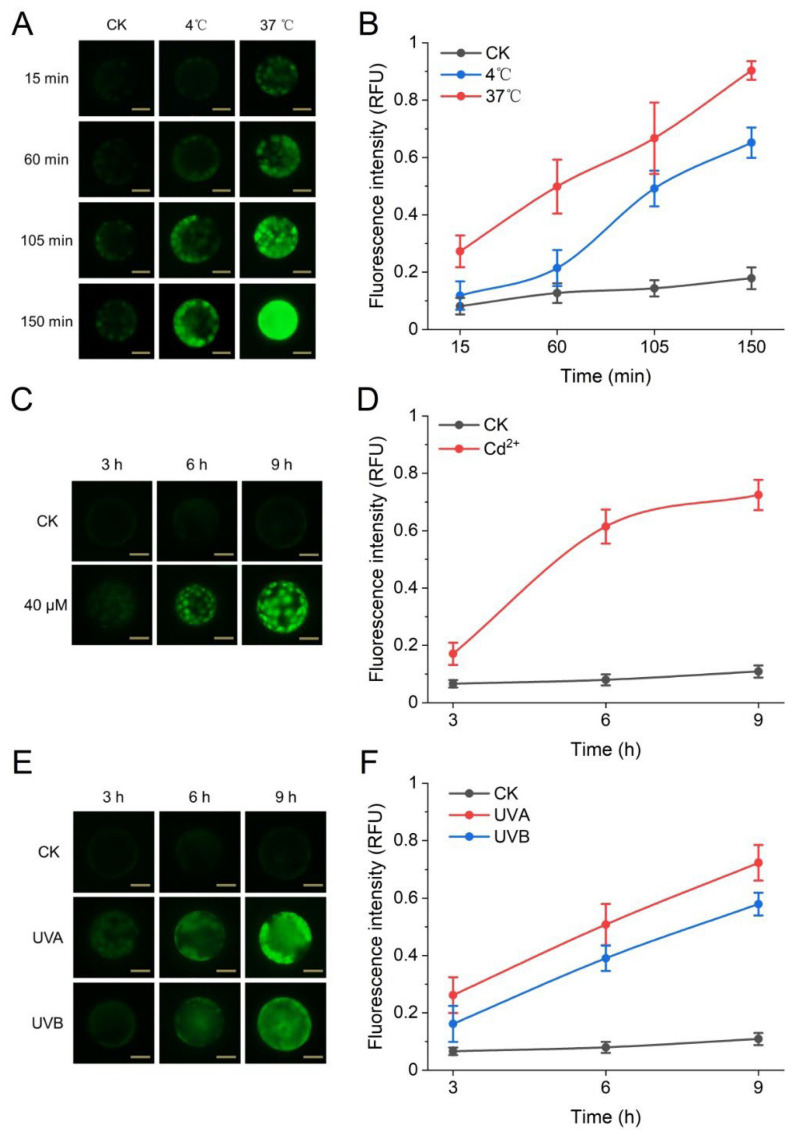
Redox status of Arabidopsis protoplasts under environmental stress. (**A**) Fluorescence images of protoplasts at different temperatures with a scale of 25 μm; (**B**) fluorescence intensity of protoplasts at different temperature stresses; (**C**) fluorescence images of protoplasts under Cd^2+^ treatment with a scale of 25 μm; (**D**) fluorescence intensity of protoplasts under Cd^2+^; (**E**) fluorescence images of protoplasts under UV treatment with a scale of 25 μm; (**F**) fluorescence intensity of protoplasts under UV.

ROS play an essential role in the physiological and developmental processes of plant growth, as well as in the defense-signal cascade against abiotic and biotic stress factors. Using the developed system, we investigated the intracellular ROS responses of mesophyll protoplasts to environmental stresses such as high and low temperatures, UV light, and heavy-metal exposure. First, when protoplasts were treated at low temperature (4 °C) and high temperature (37 °C) for 15–150 min, the intracellular ROS signal increased to a significantly higher level (3.5–5 folds after a 150-min exposure) than that in the control group (20 °C), and the oxidative pressure induced by high temperature was significantly higher than that induced by low temperature (Figure 3A,B). Second, Cd ions at a concentration of 40 μM induced an accumulation of ROS over a 9 h period, with such a change in cytosolic redox status occurring mainly in the first 6 h (Figure 3C,D). Third, after 9 h of UV-A or UV-B irradiation, the increase in ROS in two populations of mesophyll protoplasts increased linearly with time and showed almost parallel trends, with the UV-A response being stronger than the UV-B response (Figure 3E,F). A comparison of the three treatments showed that each stress has its own unique induction kinetics in the accumulation of reactive oxygen species in single plant cells. In addition, treatment with ascorbate (AsA), a free-radical scavenger, effectively suppressed the production of ROS induced by the above three treatments, suggesting the fluorescence signal detected by the system was indeed from the oxidation of protoplasts (Figure S1, Supplementary Materials). In summary, these results confirm that our system can be used for the relative quantification of biochemical and physiological properties in protoplasts in a high-throughput manner and for comparison between cell populations by selecting appropriate probes.

### 3.3. ROS Accumulation in Petal Cells Is Associated with Anthocyanin Level under UV-B Irradiation

**Figure 4:**
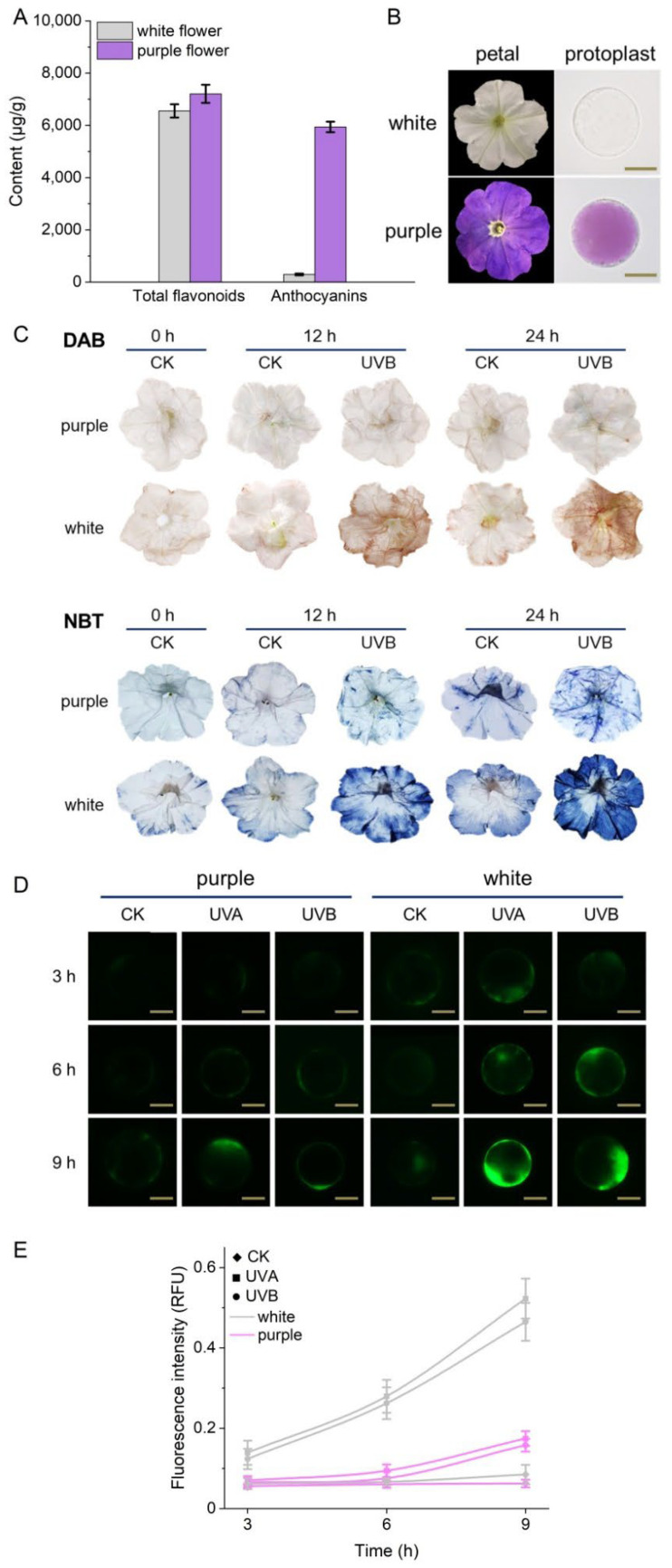
Response of Petunia protoplasts to UV irradiation. (**A**) Total flavonoid and anthocyanin content in different colored petals; (**B**) images of petals at tissue and cell levels; (**C**) DAB and NBT staining of different colored petals under UV irradiation; (**D**) fluorescence images of protoplasts ROS with a scale of 25 μm; (**E**) fluorescence intensity of different colored protoplasts on UV.

UV light is thought to cause oxidative damage in living organisms [37]. In cultured mammalian cells, UV light has been shown to stimulate H_2_O_2_ production by photoreduction in peroxisomes and mitochondria [38]. In plants, strong UV irradiation also leads to the overproduction of ROS in chloroplasts and mitochondria, which in turn has profound effects on other organelles such as peroxisomes, cytosol and vacuoles [39]. To protect themselves from UV stress, plants biosynthesize a variety of specialized metabolites in specific intracellular compartments. However, the functional characterization of these natural products is often hampered by their low abundance and limited availability in plant tissues [40], so single-cell analysis based on microfluidics can be an effective alternative tool. In this study, we are interested in the possible role of anthocyanins as part of the complex antioxidant defense system that the plant employs to minimize oxidative damage caused by UV radiation. To this end, we first measured the total flavonoid and anthocyanin content in the white and purple petals of Petunia (*Petunia hybrida*), respectively. The data showed that the purple petals had a 20-fold higher concentration of anthocyanins than the white petals, but a similar number of total flavonoids (Figure 4A). Flavonoids are classified into chalcones, flavanones, flavonols, flavones, isoflavones, 3-deoxyflavonoids, and (pro)anthocyanidins [41], and usually accumulate in vacuoles or the cell wall [42]. Due to their absorption in the UV range, flavonoids are thought to act as sunscreens. In addition, flavonoids also act as ROS scavengers due to the presence of phenolic hydroxyl groups in their structure [41]. In this work, Petunia flowers of different colors were first irradiated with UV light and then stained with either diaminobenzidine (DAB) for H_2_O_2_ accumulation [43], or nitroblue tetrazolium (NBT) for superoxide radical (O_2_^•−^) accumulation [44]. In both assays, significant differences in staining intensity were observed between the purple and white flowers (Figure 4C), suggesting that the purple flower tissue accumulates much less ROS than their white counterparts. We then isolated protoplasts from the two types of petals and again used DCFH-DA dye to measure their respective intracellular concentrations of ROS after UV-A and UV-B irradiation (Figure 4D,E). Consistent with the tissue-level results, both the white and purple protoplasts showed a time-dependent increase in ROS compared with the respective untreated control cells, but much lower concentrations of ROS were detected in the purple protoplasts compared with the white protoplasts isolated from petals, consistent with the photoprotective H_2_O_2_ scavenging effect of anthocyanins (Figure 4D,E). The preincubation of protoplasts with AsA in the dark prior to UV treatment dramatically reduced the oxidative burst, suggesting that AsA enters cells and reduces UV-induced ROS accumulation (Figure S1). The photoprotective effect of anthocyanins is thought to be due to their UV-shielding function [42] and their ability to act as effective scavengers of ROS [45]. Although further studies are needed to elucidate the mechanism of vacuole-localized anthocyanins in scavenging ROS in the cytoplasm, our data clearly demonstrate the key role of anthocyanins in the dynamic regulation of intracellular redox status under UV light overexposure at the cellular level. This heterogeneity of cellular responses also provides clues for the real-time phenotypic in situ identification and classification of subpopulations of cells under specific stimuli.

### 3.4. Auxin in the Regulation of Primary Cell Wall Regeneration Process of Protoplasts

**Figure 5:**
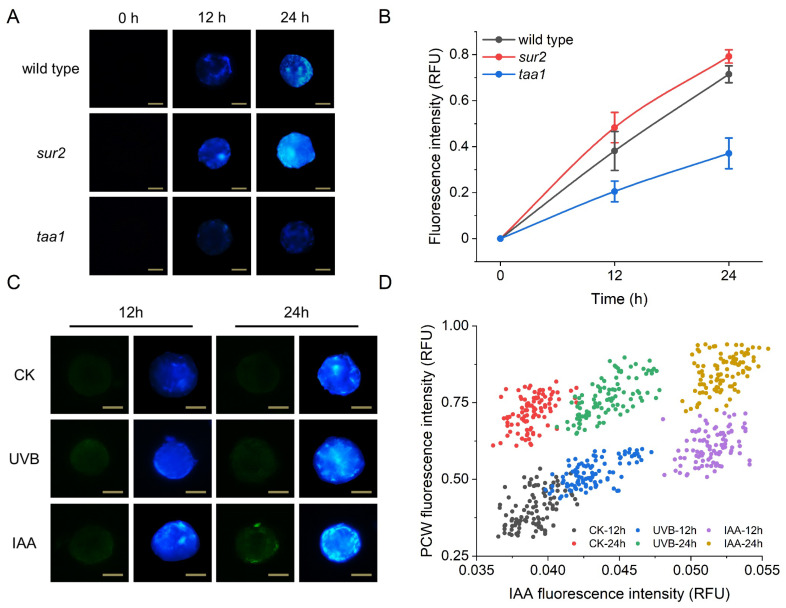
Involvement of auxin in PCW regeneration progress. (**A**) PCW fluorescence images of protoplasts of different Arabidopsis genotypes at three time points with a scale bar of 25 μm; (**B**) Fluorescence intensity of protoplasts of different Arabidopsis genotypes; (**C**) IAA distribution and PCW fluorescence images of *DRF-GFP* under the influence of UVB irradiation and exogenous IAA; (**D**) Two-channel detection of IAA autofluorescence and PCW fluorescence from *DRF-GFP* protoplasts.

In higher plants, the primary cell wall (PCW) is a highly organized structure consisting of crystalline cellulose microfibrils embedded in a hydrated matrix of pectin and hemicellulose [9]. The primary cell wall was stripped during protoplast production and will regenerate over the next 24 to 48 h [46]. Next, we investigated the physiological role of auxin under normal or UV-B conditions in the process of primary cell wall regeneration. Auxin is an essential plant hormone that plays a crucial role in many physiological and developmental processes at the cellular, tissue, and organ levels [47,48,49]. To further investigate the influence of endogenous auxin on plant cell wall regeneration, we first compared the process of primary cell wall regeneration in protoplasts of *sur2* and *taa1* mutants, which had higher and lower auxin levels [50,51], respectively, with that of wild-type mesophyll cells. Cell wall digestion and regeneration were confirmed by Calcofluor White (CFW) that specifically stains cellulose in the cell wall [30]. As shown in Figure 5, the process of cell wall regeneration was enhanced and delayed, respectively, in these two mutants (Figure 5A,B), suggesting that auxin is required for PCW regeneration of protoplasts. As an endogenous auxin, the intracellular concentration of indole-3-acetic acid (IAA) depends on its influx and efflux mediated by separate membrane-transport processes, and it has been reported that, under normal conditions, 100 pg of IAA is present in one mg of Arabidopsis root protoplast and very little IAA escapes from isolated protoplasts [52]. To monitor intracellular auxin levels, we isolated mesophyll protoplasts from the Arabidopsis *DR5:GFP* marker line, in which the IAA-responsive promoter DR5 drives fluorescent protein (GFP) expression, allowing indirect quantification of IAA within the cell [53]. As shown in Figure 5C, externally applied IAA effectively increased blue fluorescence emitted from the PCW compared with untreated protoplasts, which was accompanied by a stronger green fluorescence signal from the auxin signal reporter DR5, indicating that external auxin molecules entered the cell through the plasma membrane (Figure 5C). Considering that UV radiation causes oxidative damage to plant protoplasts, it is reasonable to assume that cells protect themselves against this damage by accelerating the formation of the protective PCW. To test this hypothesis, we irradiated *DR5:GFP* protoplasts with UV-B light and found that such treatment indeed accelerated the regeneration of PCW formation, which was underlined by a concomitant increase in auxin concentration within the cells (Figure 5C), confirming the regulatory involvement of auxin in this process. Therefore, dual-color fluorescence can be used to simultaneously analyze different physiological processes by monitoring two signals originating from the same cell. As shown in Figure 5D, six different cell clusters can be identified after the cells were grouped based on their dual-channel detection signals. Among them, the addition of exogenous auxin resulted in a higher concentration of auxin within the cell than UV-B exposure, and the promoting effect of UV-B on primary cell wall synthesis occurred mainly in the first 12 h, and at a time point of 24 h, there was little difference, probably due to the completion of the PCW in both groups. Based on the results of a large number of cells, such a small and dynamic difference was more reliable and meaningful than evaluation by microscopic observation (Figure 5D).

## 4. Summary

In this work, we have developed a microfluidic method for the rapid, efficient, and direct measurement of targeted cellular responses in protoplasts that provides an automated, easy-to-use tool for cytochemical research on single plant cells. We validated the applicability of this method for single-cell measurements of ROS in the presence of a variety of environmental stimuli, allowing a deeper understanding of redox dynamics in vivo during oxidative stress. We then applied this tool to the analysis of two different plant-specific physiological processes, and provided evidence for the photoprotective role of anthocyanin localized in the vacuole and for the formation of the primary cell wall promoted by auxin at the cellular level. The platform we developed is simple, fast, and has a high-throughput capacity. In the future, quantitative, rather than qualitative, measurements of internal biophysical and biochemical parameters of plant cells need to be further developed. By integrating electrical, optical, and magnetic sensing techniques with microfluidic technology, micro-flow cytometers are certain to provide further insight into long-standing and pressing questions in plant science.

## Supplementary Materials

The following supporting information can be downloaded at: https://www.mdpi.com/article/10.3390/bios12090688/s1, Figure S1: Fluorescence images of Arabidopsis protoplasts with and without ASA under different external stress treatments (with a scale bar of 25 μm).
