# Supplementary material for: Functional Characterization and Phenotyping of Protoplasts on a Microfluidics-Based Flow Cytometry
Source: Biosensors (Basel). 2022 Aug 26;12(9):688. doi: 10.3390/bios12090688 (PMC9496511; doi:10.3390/bios12090688)
Supplement: Supplementary file 1 [file biosensors-12-00688-s001.zip › biosensors-1862869-supplementary.pdf]

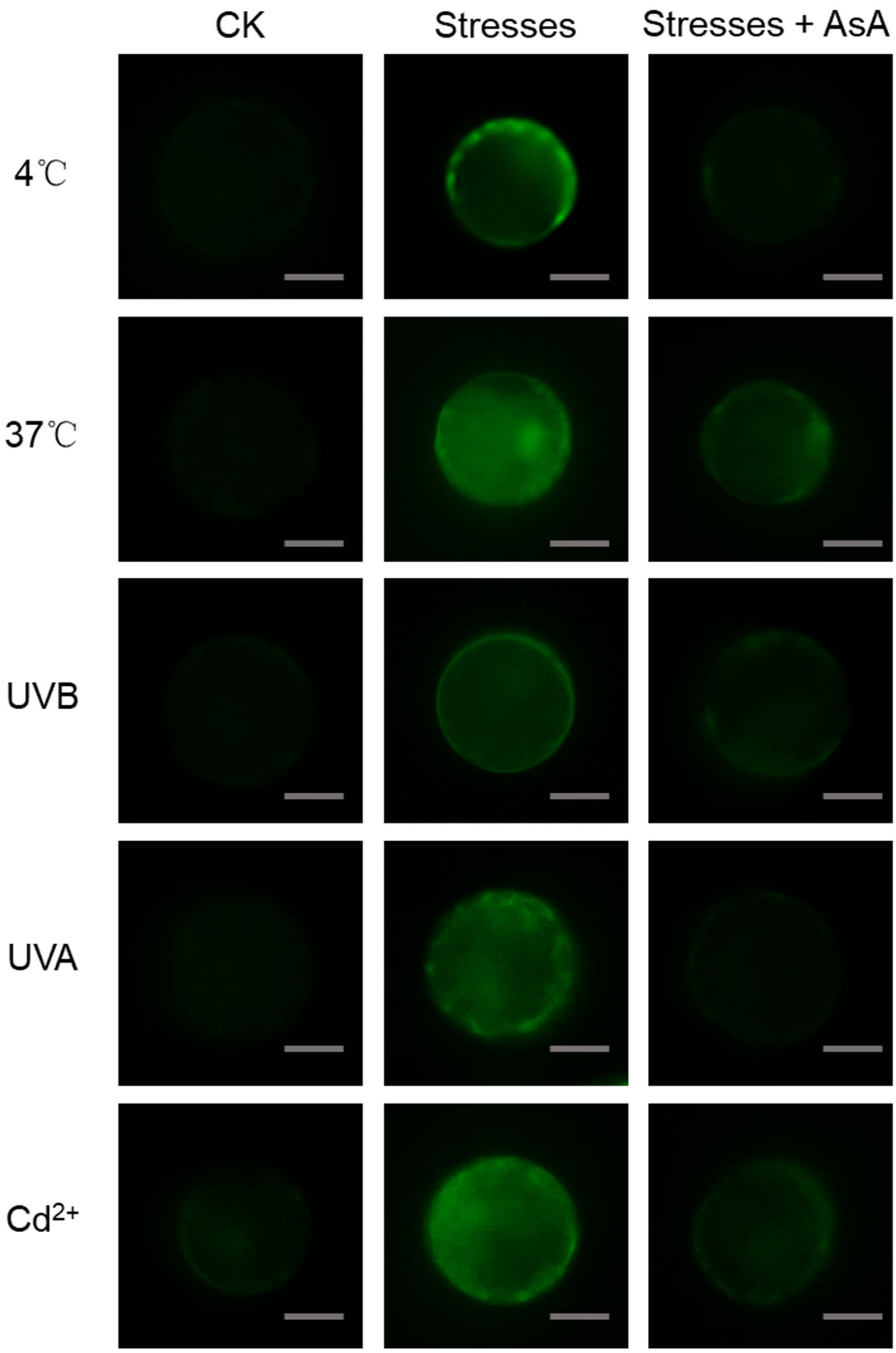

Figure S1. Fluorescence images of Arabidopsis protoplasts with and without ASA under different external stress treatments (with a scale bar of 25um).
